# Bis(2-amino-5-methyl-1,3,4-thia­diazole-κ*N* ^3^)dichloridocobalt(II)

**DOI:** 10.1107/S1600536812020995

**Published:** 2012-05-16

**Authors:** Ye Song, Yu-Fei Ji, Min-Yan Kang, Zhi-Liang Liu

**Affiliations:** a College of Chemistry and Chemical Engineering, Inner Mongolia University, Hohhot, People’s Republic of China

## Abstract

In the monomeric title complex, [CoCl_2_(C_3_H_5_N_3_S)_2_], the Co^II^ atom is tetra­coordinated by two chloride anions and two N atoms from two monodentate 2-amino-5-methyl-1,3,4-thia­diazole ligands, giving a slightly distorted tetra­hedral stereochemistry [bond angle range about Co = 105.16 (12)–112.50 (10)°]. In the complex, the dihedral angle between the 1,3,4-thia­diazole planes in the two ligands is 72.8 (1)°. There are two intra­molecular N—H⋯Cl inter­actions in the complex unit, while in the crystal, inter­molecular N—H⋯N and N—H⋯Cl hydrogen bonds link these units into a two-dimensional layered structure parallel to (011).

## Related literature

For potential applications of complexes containing 2,5-disubstituted 1,3,4-thia­diazo­les, see: Katritzky *et al.* (2010 ▶); Seed *et al.* (2007 ▶). For the preparation of the 2-amino-5-methyl-1,3,4-thia­diazole ligand, see: Chubb & Nissenbaum (1959 ▶). For complexes with this ligand, see: Lynch & Ewington (2001 ▶); Neverov *et al.* (1986 ▶); Antolini *et al.* (1988 ▶).
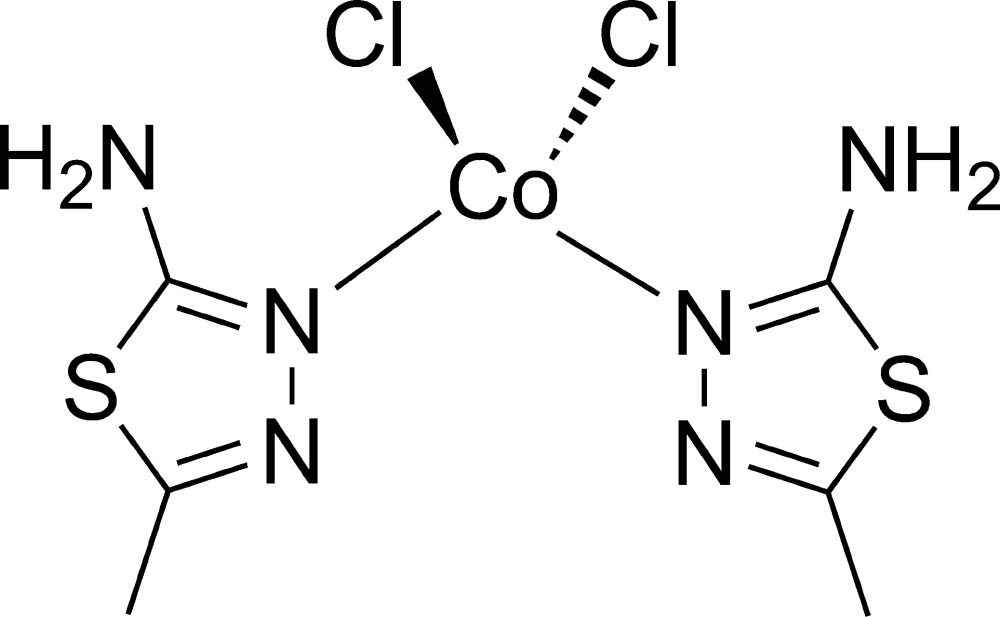


## Experimental

### 

#### Crystal data

[CoCl_2_(C_3_H_5_N_3_S)_2_]
*M* *_r_* = 360.15
Monoclinic, 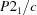

*a* = 9.124 (2) Å
*b* = 20.180 (5) Å
*c* = 7.2767 (19) Å
β = 99.479 (5)°
*V* = 1321.5 (6) Å^3^
*Z* = 4
Mo *K*α radiation
μ = 2.01 mm^−1^
*T* = 296 K
0.20 × 0.20 × 0.20 mm

#### Data collection

Bruker SMART APEX diffractometer
Absorption correction: multi-scan (*SADABS*; Sheldrick, 1996 ▶) *T*_min_ = 0.670, *T*_max_ = 0.676
8978 measured reflections
3194 independent reflections
2125 reflections with *I* > 2σ(*I*)
*R*_int_ = 0.052

#### Refinement

*R*[*F* ^2^ > 2σ(*F* ^2^)] = 0.045
*wR*(*F* ^2^) = 0.110
*S* = 1.04
3194 reflections
156 parameters
H-atom parameters constrained
Δρ_max_ = 0.47 e Å^−3^
Δρ_min_ = −0.60 e Å^−3^

### 

Data collection: *SMART* (Bruker, 2001 ▶); cell refinement: *SAINT* (Bruker, 2001 ▶); data reduction: *SAINT*; program(s) used to solve structure: *SHELXS97* (Sheldrick, 2008 ▶); program(s) used to refine structure: *SHELXL97* (Sheldrick, 2008 ▶); molecular graphics: *OLEX2* (Dolomanov *et al.*, 2009 ▶); software used to prepare material for publication: *publCIF* (Westrip, 2010 ▶).

## Supplementary Material

Crystal structure: contains datablock(s) I, global. DOI: 10.1107/S1600536812020995/zs2204sup1.cif

Structure factors: contains datablock(s) I. DOI: 10.1107/S1600536812020995/zs2204Isup2.hkl

Additional supplementary materials:  crystallographic information; 3D view; checkCIF report

## Figures and Tables

**Table 1:** Hydrogen-bond geometry (Å, °)

*D*—H⋯*A*	*D*—H	H⋯*A*	*D*⋯*A*	*D*—H⋯*A*
N1—H1*A*⋯Cl1	0.86	2.49	3.281 (4)	153
N2—H2*A*⋯Cl2	0.86	2.66	3.445 (4)	152
N2—H2*B*⋯N6^i^	0.86	2.32	3.114 (5)	153
N1—H1*B*⋯Cl2^ii^	0.86	2.65	3.387 (4)	144

Symmetry codes: (i) 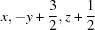; (ii) 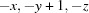.

**Fig. 1.**
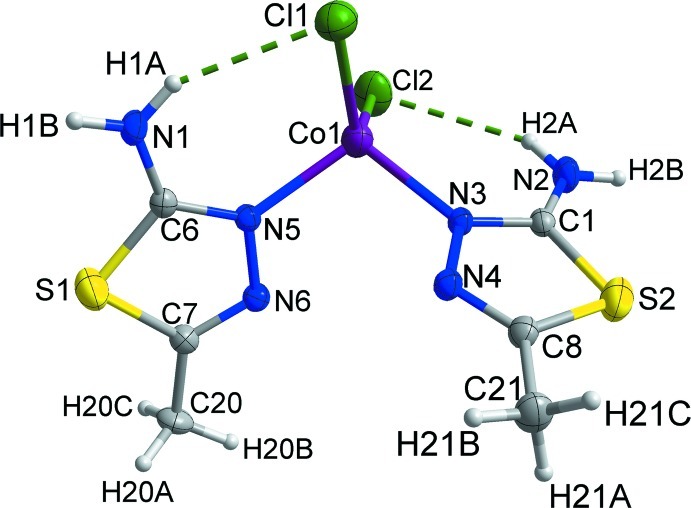
The structure and atom-numbering scheme for the title complex, with displacement ellipsoids drawn at the 30% probability level for non-H atoms. N—H···Cl interactions are shown as dashed lines.

**Fig. 2.**
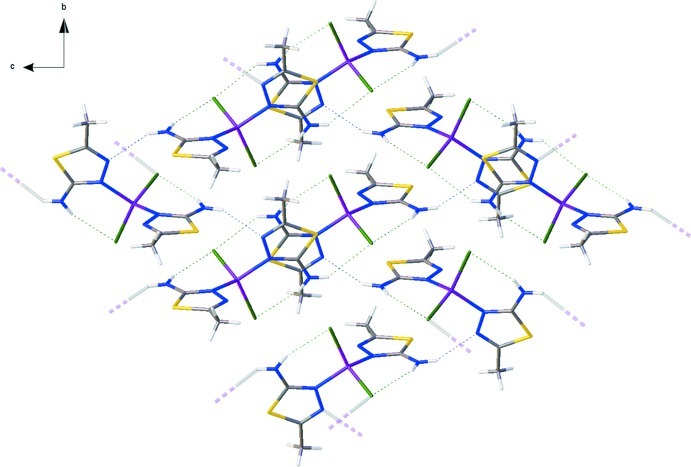
A packing diagram viewed down the a-axis of the unit cell, showing the two-dimensional layered structure.

## supplementary crystallographic information

### Comment

Recently, metal complexes with 2,5-disubstituted 1,3,4-thiadiazoles have received considerable attention, as they have a wide range of potential applications in the fields of medicine and material science (Katritzky *et al.*, 2010; Seed *et al.*, 2007). The 2-Amino-5-methyl-1,3,4-thiadiazole (amtz) ligand, which contains one S and three N coordination sites is recognized as a potential multidentate ligand to construct some interesting compounds (Lynch *et al.*, 2001; Neverov *et al.*, 1986; Antolini *et al.*, 1988). Herein, the title complex [CoCl_2_(C_3_H_5_N_3_S)_2_] has been synthesized and characterized structurally.

In the title monomeric complex, [CoCl_2_(C_3_H_5_N_3_S)_2_], the Co^II^ is tetracoordinated by two chlorine anions and two nitrogen atoms from two monodentate 2-amino-5-methyl-1,3,4-thiadiazole ligands, giving a slightly distorted tetrahedral stereochemistry [bond angle range, 105.16 (12)– 112.50 (10)°; bond lengths: Co—N = 2.004 (3) and 2.009 (3) Å; Co—Cl = 2.2416 (12) and 2.2590 (12) Å]. Since the intramolecular N—H···Cl interactions exist between the NH_2_ group and Cl anions (Table 1), the Co—Cl bonds deviate from the thiadiazole ring planes (Antolini *et al.*, 1988). In the crystal, the complex molecules are connected by intermolecular N—H···Cl and N—H···N hydrogen-bonding interactions, forming a two-dimensional layered structure which extends along the (011) plane (Fig. 2).

### Experimental

2-Amino-5-methyl-1,3,4-thiadiazole (amtz) was prepared according to a previously reported procedure (Chubb & Nissenbaum, 1959). 1 mmol of CoCl_2_ . 6H_2_O (0.237 g) dissolved in 20 ml of ethanol–water (1:1, v/v) containing 1 mmol of 2-amino-5-methyl-1,3,4-thiadiazole (0.115 g). The resulting solution was stirred continuously for about 2 h. Upon slow partial evaporation of the solvent, dark blue crystals formed after 5 days. Yield: 35% (based on CoCl_2_ . 6H_2_O).

### Refinement

All H-atoms were placed in calculated positions with N—H = 0.86 Å and C—H = 0.96 Å and were allowed to ride in the refinement, with *U*_iso_(H) = 1.2*U*_eq_(N) and *U*_iso_(H) = 1.5*U*_eq_(C).

### Crystal data

**Table:**

[CoCl_2_(C_3_H_5_N_3_S)_2_]	*F*(000) = 724
*M**_r_* = 360.15	*D*_x_ = 1.810 Mg m^−^^3^
Monoclinic, *P*2_1_/*c*	Mo *K*α radiation, λ = 0.71073 Å
Hall symbol: -P 2ybc	Cell parameters from 5643 reflections
*a* = 9.124 (2) Å	θ = 0.7–0.7°
*b* = 20.180 (5) Å	µ = 2.01 mm^−^^1^
*c* = 7.2767 (19) Å	*T* = 296 K
β = 99.479 (5)°	Block, blue
*V* = 1321.5 (6) Å^3^	0.20 × 0.20 × 0.20 mm
*Z* = 4	

### Data collection

**Table:**

Bruker SMART APEX diffractometer	3194 independent reflections
Radiation source: fine-focus sealed tube	2125 reflections with *I* > 2σ(*I*)
Graphite monochromator	*R*_int_ = 0.052
Detector resolution: 8.192 pixels mm^-1^	θ_max_ = 28.2°, θ_min_ = 2.0°
ω–2τ scans	*h* = −9→12
Absorption correction: multi-scan (*SADABS*; Sheldrick, 1996)	*k* = −26→26
*T*_min_ = 0.670, *T*_max_ = 0.676	*l* = −8→9
8978 measured reflections	

### Refinement

**Table:**

Refinement on *F*^2^	Primary atom site location: structure-invariant direct methods
Least-squares matrix: full	Secondary atom site location: difference Fourier map
*R*[*F*^2^ > 2σ(*F*^2^)] = 0.045	Hydrogen site location: inferred from neighbouring sites
*wR*(*F*^2^) = 0.110	H-atom parameters constrained
*S* = 1.04	*w* = 1/[σ^2^(*F*_o_^2^) + (0.047*P*)^2^ + 0.1324*P*] where *P* = (*F*_o_^2^ + 2*F*_c_^2^)/3
3194 reflections	(Δ/σ)_max_ < 0.001
156 parameters	Δρ_max_ = 0.47 e Å^−^^3^
0 restraints	Δρ_min_ = −0.60 e Å^−^^3^

### Special details

**Table:**

Geometry. All e.s.d.'s (except the e.s.d. in the dihedral angle between two l.s. planes) are estimated using the full covariance matrix. The cell e.s.d.'s are taken into account individually in the estimation of e.s.d.'s in distances, angles and torsion angles; correlations between e.s.d.'s in cell parameters are only used when they are defined by crystal symmetry. An approximate (isotropic) treatment of cell e.s.d.'s is used for estimating e.s.d.'s involving l.s. planes.
Refinement. Refinement of *F*^2^ against ALL reflections. The weighted *R*-factor *wR* and goodness of fit *S* are based on *F*^2^, conventional *R*-factors *R* are based on *F*, with *F* set to zero for negative *F*^2^. The threshold expression of *F*^2^ > σ(*F*^2^) is used only for calculating *R*-factors(gt) *etc*. and is not relevant to the choice of reflections for refinement. *R*-factors based on *F*^2^ are statistically about twice as large as those based on *F*, and *R*- factors based on ALL data will be even larger.

### Fractional atomic coordinates and isotropic or equivalent isotropic displacement parameters (Å^2^)

**Table:**

	*x*	*y*	*z*	*U*_iso_*/*U*_eq_	
Co1	0.15275 (5)	0.62934 (2)	0.21631 (7)	0.02936 (16)	
S2	0.52153 (11)	0.77737 (5)	0.39642 (15)	0.0386 (3)	
S1	0.26820 (12)	0.44424 (5)	−0.08418 (15)	0.0391 (3)	
Cl1	0.06975 (11)	0.58762 (5)	0.46557 (14)	0.0410 (3)	
Cl2	−0.02720 (11)	0.68410 (5)	0.02491 (16)	0.0457 (3)	
C1	0.3446 (4)	0.75138 (19)	0.3006 (5)	0.0320 (8)	
N3	0.3324 (3)	0.68679 (14)	0.2864 (4)	0.0302 (7)	
N5	0.2217 (3)	0.55309 (15)	0.0748 (4)	0.0303 (7)	
N6	0.3037 (3)	0.56944 (15)	−0.0648 (5)	0.0337 (7)	
N4	0.4654 (3)	0.65406 (15)	0.3519 (5)	0.0352 (8)	
C6	0.1944 (4)	0.48894 (18)	0.0809 (5)	0.0293 (8)	
C7	0.3353 (4)	0.5187 (2)	−0.1557 (5)	0.0336 (9)	
C8	0.5721 (4)	0.69334 (19)	0.4114 (5)	0.0355 (9)	
N1	0.1191 (4)	0.46049 (16)	0.2013 (5)	0.0434 (9)	
H1A	0.0845	0.4842	0.2825	0.052*	
H1B	0.1050	0.4183	0.1981	0.052*	
N2	0.2347 (4)	0.79357 (16)	0.2434 (5)	0.0460 (9)	
H2A	0.1488	0.7789	0.1933	0.055*	
H2B	0.2494	0.8355	0.2565	0.055*	
C20	0.4198 (5)	0.5212 (2)	−0.3141 (6)	0.0453 (11)	
H20A	0.4970	0.4884	−0.2961	0.068*	
H20B	0.4632	0.5643	−0.3198	0.068*	
H20C	0.3538	0.5125	−0.4285	0.068*	
C21	0.7265 (5)	0.6726 (2)	0.4872 (7)	0.0552 (13)	
H21A	0.7922	0.6872	0.4051	0.083*	
H21B	0.7307	0.6252	0.4976	0.083*	
H21C	0.7563	0.6920	0.6081	0.083*	

### Atomic displacement parameters (Å^2^)

**Table:**

	*U*^11^	*U*^22^	*U*^33^	*U*^12^	*U*^13^	*U*^23^
Co1	0.0291 (3)	0.0241 (3)	0.0352 (3)	−0.0010 (2)	0.0060 (2)	−0.0012 (2)
S2	0.0437 (6)	0.0292 (5)	0.0425 (6)	−0.0096 (4)	0.0060 (5)	−0.0050 (5)
S1	0.0487 (6)	0.0273 (5)	0.0421 (6)	0.0026 (4)	0.0100 (5)	−0.0051 (5)
Cl1	0.0442 (5)	0.0428 (6)	0.0381 (6)	−0.0072 (4)	0.0129 (4)	−0.0008 (5)
Cl2	0.0433 (6)	0.0360 (6)	0.0541 (7)	0.0069 (5)	−0.0029 (5)	0.0033 (5)
C1	0.039 (2)	0.0279 (19)	0.031 (2)	−0.0037 (17)	0.0099 (17)	−0.0030 (17)
N3	0.0303 (16)	0.0216 (15)	0.0387 (19)	0.0003 (13)	0.0054 (13)	0.0001 (14)
N5	0.0328 (16)	0.0271 (17)	0.0315 (18)	0.0015 (13)	0.0066 (13)	0.0005 (14)
N6	0.0374 (17)	0.0279 (17)	0.0368 (19)	−0.0001 (14)	0.0092 (14)	0.0041 (15)
N4	0.0330 (17)	0.0275 (16)	0.045 (2)	−0.0003 (14)	0.0046 (15)	0.0001 (16)
C6	0.0294 (18)	0.0277 (19)	0.029 (2)	0.0011 (15)	−0.0004 (15)	0.0013 (16)
C7	0.035 (2)	0.035 (2)	0.031 (2)	0.0025 (17)	0.0041 (16)	0.0003 (18)
C8	0.038 (2)	0.033 (2)	0.036 (2)	−0.0038 (17)	0.0065 (17)	0.0000 (18)
N1	0.057 (2)	0.0255 (17)	0.050 (2)	−0.0062 (16)	0.0168 (18)	0.0004 (16)
N2	0.047 (2)	0.0247 (17)	0.065 (3)	0.0031 (16)	0.0032 (18)	−0.0034 (18)
C20	0.044 (2)	0.058 (3)	0.037 (2)	0.008 (2)	0.0165 (19)	0.000 (2)
C21	0.041 (2)	0.054 (3)	0.067 (3)	0.003 (2)	−0.003 (2)	−0.005 (3)

### Geometric parameters (Å, º)

**Table:**

Co1—N3	2.004 (3)	N4—C8	1.275 (5)
Co1—N5	2.009 (3)	C6—N1	1.330 (5)
Co1—Cl1	2.2416 (12)	C7—C20	1.490 (5)
Co1—Cl2	2.2590 (12)	C8—C21	1.486 (5)
S2—C1	1.731 (4)	N1—H1A	0.8600
S2—C8	1.756 (4)	N1—H1B	0.8600
S1—C6	1.725 (4)	N2—H2A	0.8600
S1—C7	1.735 (4)	N2—H2B	0.8600
C1—N3	1.311 (5)	C20—H20A	0.9600
C1—N2	1.329 (5)	C20—H20B	0.9600
N3—N4	1.395 (4)	C20—H20C	0.9600
N5—C6	1.320 (4)	C21—H21A	0.9600
N5—N6	1.397 (4)	C21—H21B	0.9600
N6—C7	1.277 (5)	C21—H21C	0.9600
			
N3—Co1—N5	105.16 (12)	N6—C7—S1	114.7 (3)
N3—Co1—Cl1	112.50 (10)	C20—C7—S1	120.9 (3)
N5—Co1—Cl1	107.63 (9)	N4—C8—C21	125.1 (4)
N3—Co1—Cl2	110.78 (9)	N4—C8—S2	113.7 (3)
N5—Co1—Cl2	108.41 (9)	C21—C8—S2	121.2 (3)
Cl1—Co1—Cl2	111.99 (5)	C6—N1—H1A	120.0
C1—S2—C8	87.17 (18)	C6—N1—H1B	120.0
C6—S1—C7	87.32 (18)	H1A—N1—H1B	120.0
N3—C1—N2	124.2 (3)	C1—N2—H2A	120.0
N3—C1—S2	113.2 (3)	C1—N2—H2B	120.0
N2—C1—S2	122.5 (3)	H2A—N2—H2B	120.0
C1—N3—N4	112.7 (3)	C7—C20—H20A	109.5
C1—N3—Co1	130.6 (3)	C7—C20—H20B	109.5
N4—N3—Co1	116.2 (2)	H20A—C20—H20B	109.5
C6—N5—N6	112.5 (3)	C7—C20—H20C	109.5
C6—N5—Co1	131.1 (3)	H20A—C20—H20C	109.5
N6—N5—Co1	116.2 (2)	H20B—C20—H20C	109.5
C7—N6—N5	112.4 (3)	C8—C21—H21A	109.5
C8—N4—N3	113.2 (3)	C8—C21—H21B	109.5
N5—C6—N1	124.5 (4)	H21A—C21—H21B	109.5
N5—C6—S1	113.1 (3)	C8—C21—H21C	109.5
N1—C6—S1	122.4 (3)	H21A—C21—H21C	109.5
N6—C7—C20	124.3 (4)	H21B—C21—H21C	109.5
			
C8—S2—C1—N3	0.3 (3)	C6—N5—N6—C7	−0.4 (4)
C8—S2—C1—N2	−178.0 (4)	Co1—N5—N6—C7	−175.9 (3)
N2—C1—N3—N4	178.2 (3)	C1—N3—N4—C8	−0.3 (5)
S2—C1—N3—N4	−0.1 (4)	Co1—N3—N4—C8	−173.4 (3)
N2—C1—N3—Co1	−10.0 (6)	N6—N5—C6—N1	179.7 (3)
S2—C1—N3—Co1	171.75 (19)	Co1—N5—C6—N1	−5.7 (6)
N5—Co1—N3—C1	145.3 (3)	N6—N5—C6—S1	0.2 (4)
Cl1—Co1—N3—C1	−97.9 (3)	Co1—N5—C6—S1	174.85 (18)
Cl2—Co1—N3—C1	28.3 (4)	C7—S1—C6—N5	0.0 (3)
N5—Co1—N3—N4	−43.2 (3)	C7—S1—C6—N1	−179.5 (3)
Cl1—Co1—N3—N4	73.7 (3)	N5—N6—C7—C20	179.1 (3)
Cl2—Co1—N3—N4	−160.1 (2)	N5—N6—C7—S1	0.3 (4)
N3—Co1—N5—C6	137.7 (3)	C6—S1—C7—N6	−0.2 (3)
Cl1—Co1—N5—C6	17.5 (4)	C6—S1—C7—C20	−179.0 (3)
Cl2—Co1—N5—C6	−103.8 (3)	N3—N4—C8—C21	−179.3 (4)
N3—Co1—N5—N6	−47.8 (3)	N3—N4—C8—S2	0.6 (4)
Cl1—Co1—N5—N6	−168.0 (2)	C1—S2—C8—N4	−0.5 (3)
Cl2—Co1—N5—N6	70.7 (2)	C1—S2—C8—C21	179.4 (4)

### Hydrogen-bond geometry (Å, º)

**Table:**

*D*—H···*A*	*D*—H	H···*A*	*D*···*A*	*D*—H···*A*
N1—H1*A*···Cl1	0.86	2.49	3.281 (4)	153
N2—H2*A*···Cl2	0.86	2.66	3.445 (4)	152
N2—H2*B*···Cl1^i^	0.86	2.90	3.331 (4)	113
N2—H2*B*···N6^ii^	0.86	2.32	3.114 (5)	153
N1—H1*B*···Cl2^iii^	0.86	2.65	3.387 (4)	144
N1—H1*A*···Cl1^iv^	0.86	2.88	3.341 (3)	115

Symmetry codes: (i) *x*, −*y*+3/2, *z*−1/2; (ii) *x*, −*y*+3/2, *z*+1/2; (iii) −*x*, −*y*+1, −*z*; (iv) −*x*, −*y*+1, −*z*+1.
